# Correction to: Reducing chemotherapy use in clinically high-risk, genomically low-risk pN0 and pN1 early breast cancer patients: five-year data from the prospective, randomised phase 3 West German Study Group (WSG) PlanB trial

**DOI:** 10.1007/s10549-018-05105-8

**Published:** 2019-01-10

**Authors:** Ulrike Nitz, Oleg Gluz, Matthias Christgen, Ronald E. Kates, Michael Clemens, Wolfram Malter, Benno Nuding, Bahriye Aktas, Sherko Kuemmel, Toralf Reimer, Andrea Stefek, Fatemeh Lorenz-Salehi, Petra Krabisch, Marianne Just, Doris Augustin, Cornelia Liedtke, Calvin Chao, Steven Shak, Rachel Wuerstlein, Hans H. Kreipe, Nadia Harbeck

**Affiliations:** 1 West German Study Group, Ludwig-Weber-Str. 15, 41061 Moenchengladbach, Germany; 2 Ev. Hospital Bethesda, Breast Center Niederrhein, Ludwig-Weber-Str. 15, 41061 Moenchengladbach, Germany; 3 Institute of Pathology, Medical School Hannover, Carl-Neuberg-Str. 1., 30625 Hannover, Germany; 4 Department of Oncology, Clinics Mutterhaus der Borromäerinnen, Feldstraße 16, 54290 Trier, Germany; 5 Department of Gynecology and Obstetrics, Breast Center, University Clinics Cologne, Kerpener Str. 62, 50937 Cologne, Germany; 6 Department of Gynecology and Obstetrics, Evangelical Hospital, Ferrenbergstraße 24, 51465 Bergisch Gladbach, Germany; 7 Department of Gynecology and Obstetrics, University Clinics Essen, Hufelandstraße 55, 45147 Essen, Germany; 8 Clinics Essen-Mitte, Breast Centre, Henricistraße 92, 45136 Essen, Germany; 9 Department of Gynecology and Obstetrics, Clinics Suedstadt, Südring 81, 18059 Rostock, Germany; 10 Johanniter Clinics Stendal, Breast Center Altmark, Bahnhofstraße 24 – 26, 39576 Stendal, Germany; 11 Department of Gynecology, Dr. Horst-Schmidt Clinics, Ludwig-Erhard-Straße 100, 65199 Wiesbaden, Germany; 12 Klinikum ChemnitzFlemmingstraße 2, 09116 Chemnitz, Germany; 13 Oncological Practice, Teutoburger Str. 60 33604, Bielefeld, Germany; 14 Breast Centre Ostbayern, Perlasberger Str. 41, 94469 Deggendorf, Germany; 15 Department of Gynecology and Obstetrics, University Hospital Schleswig–Holstein Campus Luebeck, Ratzeburger Allee 160, 23562 Luebeck, Germany; 16 Genomic Health, Inc., Redwood City, CA USA; 17 Department of Gynecology and Obstetrics, Breast Center, University of Munich (LMU) and CCCLMU, Munich, Germany

## Correction to: Breast Cancer Res Treat (2017) 165:573–583 10.1007/s10549-017-4358-6

The article Reducing chemotherapy use in clinically high-risk, genomically low-risk pN0 and pN1 early breast cancer patients: five-year data from the prospective, randomised phase 3 West German Study Group (WSG) PlanB trial, written by Ulrike Nitz, Oleg Gluz, Matthias Christgen, Ronald E. Kates, Michael Clemens, Wolfram Malter, Benno Nuding, Bahriye Aktas, Sherko Kuemmel, Toralf Reimer, Andrea Stefek, Fatemeh Lorenz-Salehi, Petra Krabisch, Marianne Just, Doris Augustin, Cornelia Liedtke, Calvin Chao, Steven Shak, Rachel Wuerstlein, Hans H. Kreipe, Nadia Harbeck, was originally published electronically on the publisher’s internet portal (currently SpringerLink) on June 29, 2017 without open access.With the author(s)’ decision to opt for Open Choice the copyright of the article changed on January 6, 2019 to © The Author(s) 2017 and the article is forthwith distributed under the terms of the Creative Commons Attribution-NonCommercial 4.0 International License (http://creativecommons.org/licenses/by-nc/4.0/), which permits any noncommercial use, duplication, adaptation, distribution and reproduction in any medium or format, as long as you give appropriate credit to the original author(s) and the source, a link is provided to the Creative Commons license and any changes made are indicated. The original article has been corrected.

